# Supplementary material for: Two-signal requirement for growth-promoting function of Yap in hepatocytes
Source: eLife. 2015 Feb 10;4:e02948. doi: 10.7554/eLife.02948 (PMC4363878; doi:10.7554/eLife.02948)
Supplement: Supplementary file 1. — List of primer sequences. DOI: http://dx.doi.org/10.7554/eLife.02948.031 [file elife02948s001.docx]

Primer list

| YapKI DNA recombination qPCR | |
| --- | --- |
| KI-geno-f | AAAGTCGCTCTGAGTTGTTAT |
| KI-geno-r1 | AAGACCGCGAAGAGTTTGTC |
| YapKO DNA recombination qPCR | |
| KO-q-f | ACTCAAAAACGGGGTTTTCC |
| KO-q-r | TTGAAGCTCCCTCGAAAGG |
| DNA recombination qPCR normalizer | |
| Rag2-f | TAAGAGTGGACCTTCCCCTG |
| Rag2-r | ATTTCAATCGTGTTGTCCCC |
| Genotyping | |
| YapKO genotyping | |
| KO-geno-f | CCCATGTTTGTGTCCATCTG |
| KO-geno-r | TCTTCACATTTTGAGGGGAAA |
| YapKI Genotyping | |
| KI-geno-f | AAAGTCGCTCTGAGTTGTTAT |
| KI-geno-r1 | AAGACCGCGAAGAGTTTGTC |
| KI-geno-r2 | GGAGCGGGAGAAATGGATATG |
| qPCR of mRNA targets | |
| Yap qPCR | |
| Total Yap | |
| Yap-f | gaattagctctgcgcagccag |
| Yap-r | catccacactgttgaggaagtcg |
| Endogenous Yap | |
| Yap-endo-f | GCATGAGCAGCTACAGCATC |
| Yap-endo-r | GCGTGCACATCTTAGGCTCT |
| Exogenous Yap | |
| Yap-exo-f | CAAGGATGACGATGACaagC |
| Yap-exo-r | TTCATGACGGCATTGAAGAG |
| Ctgf | |
| mCtgf-qPCR-f | CCACCCGAGTTACCAATGAC |
| mCtgf-qPCR-r | GACAGGCTTGGCGATTTTAG |
| Bcl2 | |
| mBcl2-qPCR-f | GGTGGTGGAGGAACTCTTCA |
| mBcl2-qPCR-r | CATGCTGGGGCCATATAGTT |
